# Phosphoinositide‐dependent Kinase‐1 (PDPK1) regulates serum/glucocorticoid‐regulated Kinase 3 (SGK3) for prostate cancer cell survival

**DOI:** 10.1111/jcmm.15876

**Published:** 2020-09-14

**Authors:** Geetha Nalairndran, Azad Hassan Abdul Razack, Chun‐Wai Mai, Felicia Fei‐Lei Chung, Kok‐Keong Chan, Ling‐Wei Hii, Wei‐Meng Lim, Ivy Chung, Chee‐Onn Leong

**Affiliations:** ^1^ Department of Pharmacology Faculty of Medicine University of Malaya Kuala Lumpur Malaysia; ^2^ Department of Surgery Faculty of Medicine University of Malaya Kuala Lumpur Malaysia; ^3^ Center for Cancer and Stem Cell Research Institute for Research Development and Innovation (IRDI) International Medical University Kuala Lumpur Malaysia; ^4^ School of Pharmacy International Medical University Kuala Lumpur Malaysia; ^5^ Mechanisms of Carcinogenesis Section (MCA) Epigenetics Group (EGE) International Agency for Research on Cancer World Health Organization Lyon France; ^6^ School of Medicine International Medical University Kuala Lumpur Malaysia; ^7^ School of Postgraduate Studies International Medical University Kuala Lumpur Malaysia; ^8^ Faculty of Medicine University of Malaya Cancer Research Institute University of Malaya Kuala Lumpur Malaysia

**Keywords:** BX‐795, GSK2334470, PDPK1, prostate cancer, RNAi screen, SGK3

## Abstract

Prostate cancer (PCa) is the most common malignancy and is the second leading cause of cancer among men globally. Using a kinome‐wide lentiviral small‐hairpin RNA (shRNA) library screen, we identified phosphoinositide‐dependent kinase‐1 (PDPK1) as a potential mediator of cell survival in PCa cells. We showed that knock‐down of endogenous human PDPK1 induced significant tumour‐specific cell death in PCa cells (DU145 and PC3) but not in the normal prostate epithelial cells (RWPE‐1). Further analyses revealed that PDPK1 mediates cancer cell survival predominantly via activation of serum/glucocorticoid‐regulated kinase 3 (SGK3). Knock‐down of endogenous PDPK1 in DU145 and PC3 cells significantly reduced SGK3 phosphorylation while ectopic expression of a constitutively active SGK3 completely abrogated the apoptosis induced by PDPK1. In contrast, no such effect was observed in SGK1 and AKT phosphorylation following PDPK1 knock‐down. Importantly, PDPK1 inhibitors (GSK2334470 and BX‐795) significantly reduced tumour‐specific cell growth and synergized docetaxel sensitivity in PCa cells. In summary, our results demonstrated that PDPK1 mediates PCa cells’ survival through SGK3 signalling and suggest that inactivation of this PDPK1‐SGK3 axis may potentially serve as a novel therapeutic intervention for future treatment of PCa.

## 1 INTRODUCTION

As the most prevalent form of non‐cutaneous cancer, prostate cancer (PCa) is the second‐highest incidence of cancer in male globally.^1^ In 2018, approximately 1.3 million new patients were diagnosed with PCa, and nearly 360 000 deaths occurred globally.^1^ In the early stages, PCa is mainly regulated by androgen, thus, androgen deprivation therapy (ADT) has become routine in clinical practice. However, about 10%‐20% of patients inevitably fail this therapy and progress to castration‐resistant PCa (CRPC) with a median survival ranges between 15 and 36 months.^2^

Despite the fact that our understanding of the clinical, molecular and pathologic characteristics of PCa is incomplete, the androgen receptor (AR), which is regarded as the primary oncoprotein in PCa and CRPC, is regularly expressed in a heterogeneous way, even in the context of AR gene amplification.^3^ In AR‐positive PCa, hormonal treatment resistance can arise via clonal selection, intracrine mechanisms or adaptation to decreased androgen (eg mutation, AR phosphorylation and bypass of the AR pathway).^4^

While some of the early studies indicated that CRPC depends on AR activity, numerous new evidence suggests that other mechanisms have the capability to promote CRPC progression in a manner that is independent of AR activation.^5^ For example, several studies have shown that phosphoinositide 3‐kinase (PI3K) signalling is adequate for CRPC survival when AR activity is reduced or not present.^5^, ^6^, ^7^

In contrast to primary PCa, AR gene expression signatures were found to be inversely correlated with cell proliferation signatures in a subset of CRPC patients.^8^ Furthermore, AR activities have also been shown to possess a tumour and metastasis suppressor function, suggesting PCa disease progression can be driven by AR‐independent mechanisms.^9^, ^10^

In order to identify genes and pathways that modify PCa growth in the context of suppressed AR signalling, we conducted in vitro high‐throughput RNA interference (RNAi) screening using a lentiviral‐shRNA library designed to target the whole kinome against the AR‐negative PCa DU145 cell line.^11^

Here, we describe the results of our screen that identified the 3‐phosphoinositide‐dependent protein kinase 1 (PDPK1) as an essential kinase critical for the proliferation and viability of a subset of PCa cells.

## 2 MATERIALS AND METHODS

### 2.1 Cell lines and cell cultures

The human PCa cells (DU145, PC3 and LNCaP) and normal prostate epithelial cell (RWPE‐1) were obtained from American Type Culture Collection (ATCC) (Manassas, VA, USA). DU145 and PC3 are AR‐negative while LNCaP harbours an androgen‐responsive AR mutant T877A.^11^ All PCa cells were maintained in RPMI 1640 supplemented with 10% foetal bovine serum (FBS) and 1% penicillin/streptomycin (Sigma‐Aldrich, St. Louis, MO, USA). The RWPE‐1 normal prostate cell line was grown in keratinocyte serum‐free medium consisting of 5 ng/mL of recombinant epidermal growth factor and 0.05 mg/mL of bovine pituitary extract (Invitrogen, Carlsbad, CA, USA). All cells were cultured at their logarithmic growth in a humidified 37°C, 5% CO_2_ incubator.

### 2.2 Lentiviral human kinase shrna library screen

The MISSION LentiExpress™ Human Kinases shRNA library (Sigma‐Aldrich, St Louis, MO, USA) was used to screen for candidate protein kinases mediating the growth of PCa cells. Briefly, the AR‐negative DU145 cells were seeded in a 384‐well plate overnight, followed by transduction of lentiviral particles at multiplicities of infection (MOI) of 1 in the presence of 7.5 μg/mL polybrene (Sigma‐Aldrich, St Louis, MO, USA). After 18h incubation, the medium containing the lentivirus particles was replaced with complete medium, and the cell viability was evaluated using the CellTiter‐Glo® assay (Promega, Madison, WI, USA) at 72 hours post‐transduction. Lentiviral particles carrying an empty vector (pLKO.1‐puro), a non‐target shRNA (NS) or a GFP expressing lentiviral construct were included as controls to examine transduction efficiency and well‐to‐well variation. All data were normalized against NS controls and hits were considered when shRNA targeting a specific gene achieved a *Z*‐score of less than −2.^12^, ^13^

### 2.3 Quantitative real‐time PCR (QPCR) analysis

The total RNA extraction and first‐strand cDNA synthesis were conducted using RNeasy Mini Kit (Qiagen, Valencia, CA, USA) and High Capacity RNA to cDNA Master Mix (Applied Biosystems, Carlsbad, CA, USA), respectively. Gene expression levels were quantified by CFX96 PCR detector system (Bio‐Rad, Richmond, CA, USA) in the presence of FastStart Universal SYBR Green Master reagent (Roche Diagnostics, Indianapolis, IN, USA). The specific forward and reverse primer sequences are shown in Table S1. The qPCR condition that applied for all samples were: 94°C for 3 minutes, followed by 40 cycles of 94°C for 40 seconds, 60°C for 40 seconds and 72°C for 25 seconds. Glyceraldehyde‐3‐phosphate dehydrogenase *(GAPDH)* was served as housekeeping gene for normalization.

### 2.4 Western blot analysis

All cell protein lysates were harvested using ice‐cold lysis buffer (1% NP‐40, 1 mM DTT, protease inhibitors, and phosphatase inhibitor I and II cocktails in PBS) as previously described.^14^, ^15^ A total of 50μg protein was loaded for immunoblotting. Monoclonal antibodies against PDPK1 and β‐actin were purchased from Santa Cruz Biotechnology, CA, USA. Primary antibodies against AR, PDPK1, p‐PDPK1 (S241), PTEN, AKT, p‐AKT (S473), p‐AKT (T308), SGK3, p‐SGK3 (T320), SGK1 and p‐SGK1 (S78) were obtained from Cell Signalling Technology, MA, USA.

### 2.5 Lentiviral production and transduction

Lentiviral non‐targeting shRNA (NS) and shRNA constructs targeting PDPK1, CAMKV and CKS1B were purchased from Sigma‐Aldrich, MO, United States, with target sequences shown in Table S2. To produce the lentiviral particles of interest, the target shRNA constructs were co‐transfected into HEK‐293T cells with lentiviral packaging plasmids, psPAX2 (Addgene plasmid #12260) and envelope plasmids, pMD2.G (Addgene plasmid #12259) as described previously.^16^, ^17^, ^18^ The lentiviral particles were then collected and added with 7.5 µg/mL polybrene (Sigma‐Aldrich, St Louis, MO, USA) for transduction.

### 2.6 Detection of apoptosis by annexin V flow cytometry

All floating and attached cells were stained for cell apoptosis assay using a PE Annexin V Apoptosis Detection Kit (BD Biosciences, San Jose, CA, USA) as described previously.^19^, ^20^ The samples were quantitated using a FACSCalibur flow cytometer and analysed by CellQuest Pro software (version 5.1.1; BD Biosciences, San Jose, CA, USA).

### 2.7 Transfection

Plasmids for constitutively active myristoylated AKT and SGK3‐S486D mutant were obtained from Addgene (Addgene plasmid # 9008) and Gene Universal (Newark, DE, USA), respectively. Plasmids were transfected into target cells using X‐tremeGENE HP DNA transfection reagent (Roche Diagnostics, Indianapolis, IN, USA) according to the manufacturer's protocol.

### 2.8 Drug combination analysis

Drug combinatory effects were analysed using the Chou‐Talalay method and Highest Single Agent (HSA) models as described previously.^21^, ^22^ Briefly, cells were plated at 2.5 × 10^3^ cells/well in 96‐well format and treated with docetaxel and/or PDPK1 inhibitors (GSK2334470 and BX795) alone or in combination. The plates were terminated by MTT cell proliferation assay at 72 hours after treatment.^23^, ^24^ Calcusyn 2.1 software (Biosoft, Cambridge, UK) was used to generate combination index (CI) based on Chou‐Talalay method,^19^, ^25^ in which CI < 1, = 1 and >1 indicates synergism, additive and antagonism effect, respectively (Table S3). The dose‐response surface curves with levels of HSA synergy were plotted by Combenefit software (Cancer Research UK Cambridge Institute).^26^

### 2.9 Statistical analysis

All results were presented as mean ± standard deviation (SD) from at least three independent experiments. SPSS (version 19.0 INC, Chicago, IL) was used to evaluate the statistical significance based on Student's independent t test. A *P*‐value <0.01 was considered statistically significant.

## 3 RESULTS

### 3.1 Kinome‐wide shRNA library screen identifies PDPK1 as putative target mediating PCa cell survival

**FIGURE 1:**
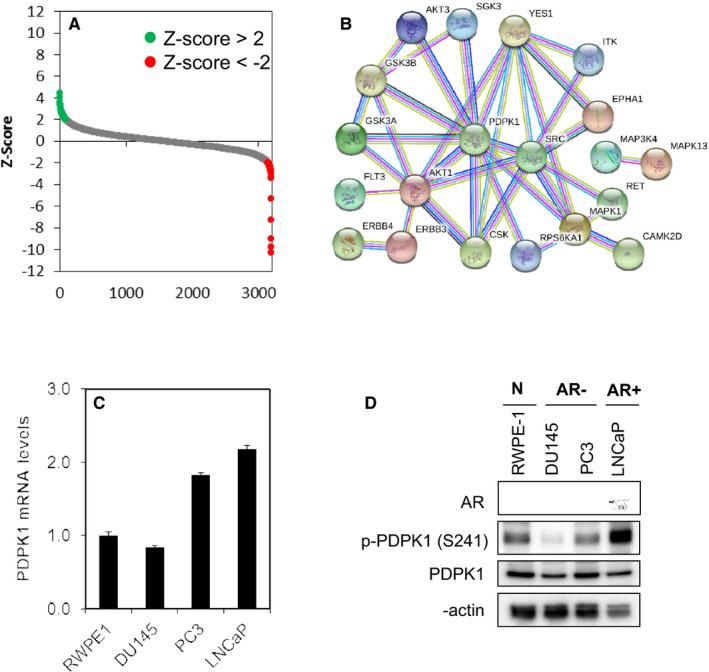
Kinome‐wide shRNA library screen identifies PDPK1 as putative target regulating the survival of PCa cells. A, Kinase shRNA screen scatter plot. Z‐scores are plotted on the y‐axis against 3109 corresponding shRNAs on the x‐axis. The red and green circled dots represent shRNA hits, which the former inhibited cell proliferation and the latter promoted cell proliferation. B, Protein‐protein interaction network of the PDPK1 target proteins. C and D, PDPK1 is expressed in a subset of PCa cells and RWPE‐1 non‐transformed prostate epithelial cells. PDPK1 mRNA expression was evaluated by qPCR with GAPDH as housekeeping gene. The level of PDPK1 protein expression was detected by immunoblotting with β‐actin as loading control

To identify genes and pathways that modify PCa growth, we conducted in vitro high‐throughput RNA interference (RNAi) screening using the RNAi Consortium (TRC) kinome shRNA library consisting of 3197 lentiviruses carrying shRNA sequences targeting 506 human kinase genes. Each gene is represented by at least 3–5 individual constructs, targeting different regions of the gene sequence. A total of 45 kinases (8.9%) in the TRC kinome library were identified to induce significant growth inhibition (Z‐score < −2) in DU145 PCa cells (Figure 1A and B, Table S4). Among them include a number of proto‐oncogene (*ERBB3*, *ERBB4*, *RET*, *SRC* and *YES1*), pro‐survival genes involved in the PI3K signalling (*AKT1*, *AKT3*, *GSK3A*, *GSK3B*, *PDPK1* and *SGK3*) and MAPK signalling (*MAP3K4*, *MAPK1*, *MAPK4*, *MAPK13* and *MAPK15*). Importantly, knock‐down of AKT1,^27^, ^28^, ^29^ AKT3,^27^, ^28^, ^29^ GSK3A,^30^, ^31^, ^32^ GSK3B,^30^, ^31^, ^32^, ^33^, ^34^, ^35^ MAPK1 (also known as ERK2),^36^, ^37^ MAPK4 (also known as ERK4)^29^ and ROCK2^38^, ^39^, ^40^ have been shown to inhibit PCa cell growth, independently validated our results.

Next, we selected 3 candidate kinases (PDPK1, CAMKV and CKS1B) for further study based on the novelty and the magnitude of effect by shRNA knock‐down. Indeed, depletion of the endogenous PDPK1, CAMKV and CKS1B significantly reduced DU145 cell survival, consistent with the results obtained in the primary screen (Figure S1).

Recent studies have shown that the locus containing PDPK1 gene (16p13.3) is more frequently amplified in lymph node metastases and castration‐resistant PCa, compared to primary tumours,^41^ we decided to focus on understanding the mechanism underlying PDPK1 mediated cell survival in PCa cells. We first evaluated whether PDPK1 is expressed in a panel of AR‐negative (DU145 and PC3) and AR‐positive (LNCaP) PCa cell lines, as well as non‐transformed prostate epithelial cells (RWPE‐1). Real‐time qPCR shows that *PDPK1* mRNA was highly expressed in all the PCa and normal prostate epithelial cells (Figure 1C). The level of gene expression correlated well with the PDPK1 protein expression as PDPK1 proteins were highly expressed in LNCaP, PC3, RWPE‐1 and DU145 (Figure 1D). Interestingly, PDPK1 proteins were found to be phosphorylated in cells which express them, suggesting that PDPK1 proteins are constitutively active in these cells.

### 3.2 Depletion of PDPK1 induces tumour‐specific cell death PCa cells

**FIGURE 2:**
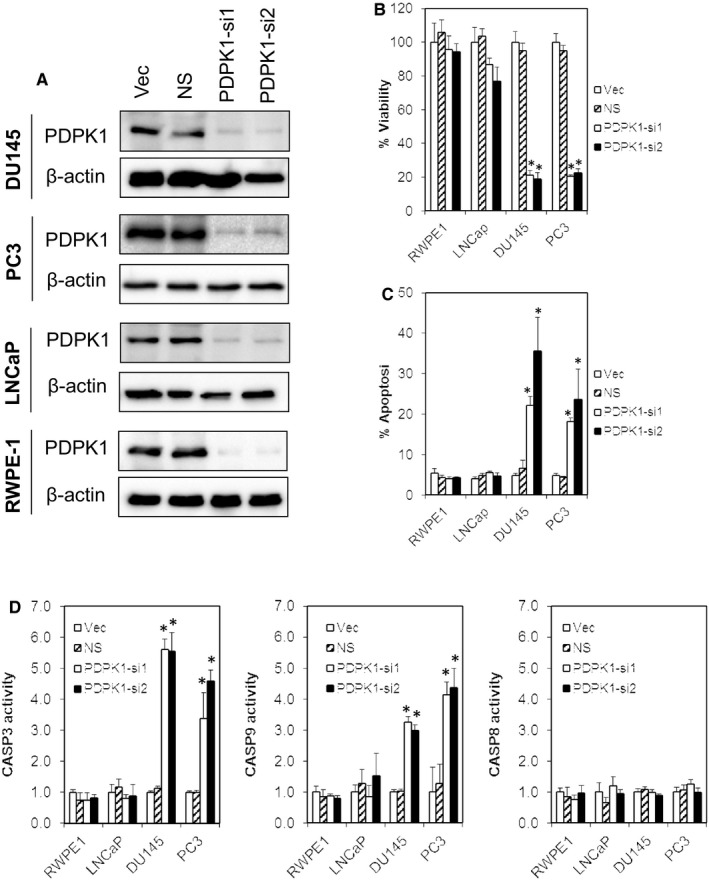
Depletion of endogenous PDPK1 induces tumour‐specific cell death in PCa cells. A, Effective PDPK1 knock‐down was achieved by two independent shRNA constructs targeting PDPK1 (PDPK1‐si1 and PDPK1‐si2). Lysates were harvested at 72 h post‐lentiviral transduction and analysed by immunoblotting. B and C, PDPK1 depletion selectively inhibited the proliferation and induced apoptosis in AR‐negative DU145 and PC3 PCa cells but not in AR‐positive LNCaP or RWPE‐1 non‐transformed prostate epithelial cells. Cell viability and apoptosis were measured using CellTiter‐Glo® assay and annexin V/7‐AAD flow cytometry at 72 h post‐transduction. D, Depletion of endogenous PDPK1 induced caspase 3 and 9 activities. Caspase 3, 8 and 9 activities were evaluated by CaspaseGlo assay at 72 h post‐transduction. Bars represent means ± SD of three independent experiments. (*) indicates statistical significance compared with NS control cells (*P* < .01, Student's t test)

To determine whether depletion of endogenous PDPK1 has any effect on the proliferation and survival of PCa cells that exhibit active PDPK1, we performed lentiviral shRNAs‐mediated knock‐down of PDPK1 in a panel of PCa and non‐transformed prostate epithelial cells. Efficient knock‐down of PDPK1 in all prostate cell lines by two independent shRNA constructs was demonstrated in Western blotting (Figure 2A). Significant reduction in cell viability of at least 80% in DU145 and PC3 was observed while no effect was observed in LNCaP and RWPE‐1 (*P* < .05, Student's t test) (Figure 2B and Figure S2). Consistent with the findings from cell viability assay, depletion of endogenous PDPK1 also induced significant amount of apoptosis in DU145 and PC3 cells (*P* < .01, Student's t test) (Figure 2C), corroborated with the induction of caspase 3 and 9, but not caspase 8 activity (Figure 2D). In contrast, no such effects were observed in RWPE‐1 nor in LNCaP cells. These results suggest that PDPK1 is required for the survival of the AR‐negative DU145 and PC3 cells, but not for the AR‐positive LNCaP cells.

### 3.3 Depletion of PDPK1 inhibits SGK3 phosphorylation

**FIGURE 3:**
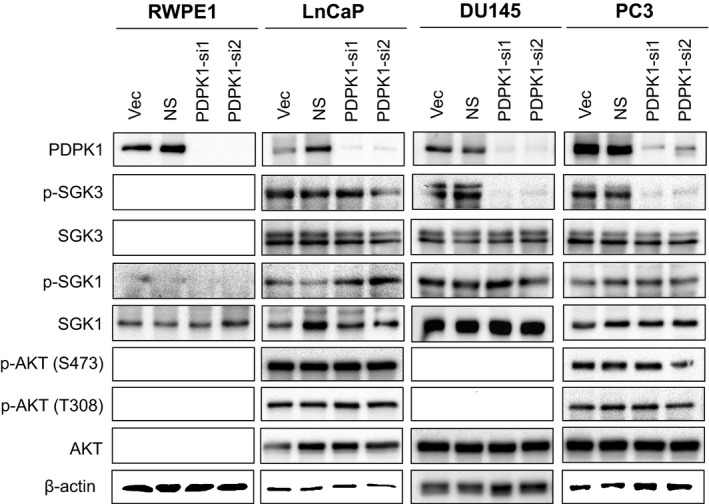
Depletion of endogenous PDPK1 reduces SGK3 phosphorylation. PDPK1 depletion down‐regulated SGK3 phosphorylation in AR‐negative DU145 and PC3 cells, but not in AR‐positive LNCaP PCa cells or RWPE‐1 non‐transformed prostate epithelial cells. The protein expression and phosphorylation of AKT, SGK1 and SGK3 Lysates were analysed by immunoblotting with β‐actin served as loading controls

PDPK1 is known to phosphorylate AKT that regulates several signalling pathways altered in cancer.^42^ However, recent studies have also shown that PDPK1 can activate many other members of AGC kinase family such as p70S6K, SGK, p90RSK and the members of PKC family, independent of AKT.^42^, ^43^, ^44^ To evaluate whether the pro‐survival effects of PDPK1 in PCa cells are mediated through aberrant activation of downstream AKT or SGK pathways, we analysed the effects of PDPK1 knock‐down on the expression and phosphorylation of these targets. As shown in Figure 3, knock‐down of PDPK1 in DU145 and PC3 cells significantly reduced phosphorylation of SGK3 but not the phosphorylation of AKT or SGK1. The total expression of SGK3, SGK1 and AKT remained unchanged. In stark contrast, no such effects were observed in RWPE‐1 or LNCaP cells.

### 3.4 PDPK1 mediates the survival of DU145 and PC3 cells via SGK3 signalling pathway

**FIGURE 4:**
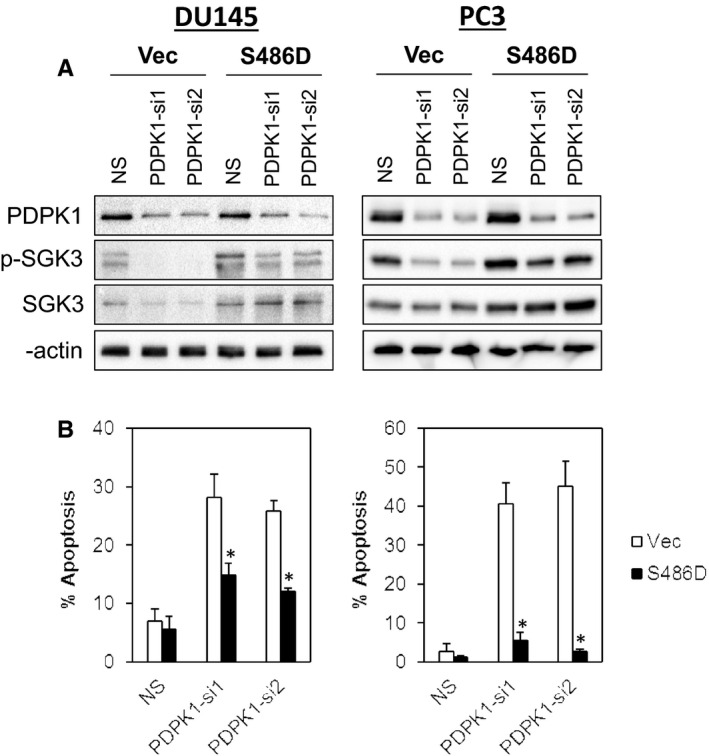
PDPK1 regulates the survival of DU145 and PC3 cells via SGK3 signalling pathway. Ectopic expression of a constitutively active SGK3 (S486D) reduced apoptotic cell death induced by PDPK1 knock‐down in DU145 cells and PC3 cells. A, The constitutively active SGK3 S486D plasmid was cotransfected with either NS or PDPK1 targeting shRNAs into DU145 and PC3 PCa cells. Whole cell protein lysates were harvested 72 h post‐transfection and analysed by immunoblotting. B, The apoptotic cell death was detected by annexin V/7‐AAD flow cytometry. Bars represent means ± SD of three independent experiments. (*) indicates statistical significance compared with Vec control cells (*P* < .01, Student's t test)

To test whether the pro‐survival effects of PDPK1 is mediated through SGK3, we transfected a constitutively active SGK3 S486D mutant in DU145 and PC3 cells followed by PDPK1 depletion. As shown in Figure 4, ectopic expression of SGK3 S486D significantly rescued the apoptosis induced by PDPK1 depletion (*P* < .01, Student's t test). In contrast, no such effects were observed in cells transfected with a constitutively active myristoylated AKT (Myr‐AKT), suggesting that PDPK1 regulates cell survival in PCa cells through activation of SGK3 signalling (Figure S3).

### 3.5 Inhibition of PDPK1 enhances docetaxel sensitivity in PCa cells

**FIGURE 5:**
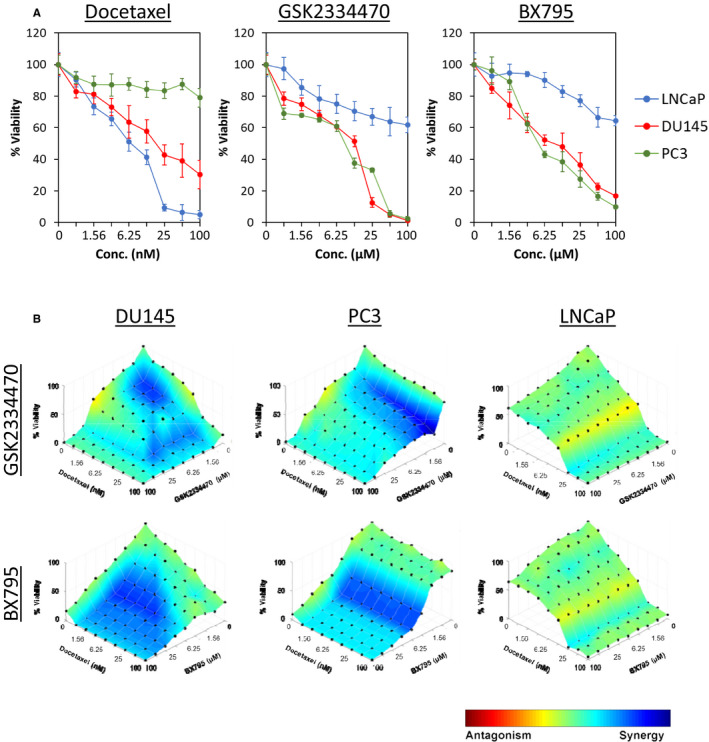
Inhibition of PDPK1 enhances docetaxel sensitivity in PCa cells. A, The PCa cells were treated with docetaxel, GSK2334470 and BX795 for 72 h, followed by evaluation of cell viability. Each data point represents mean ± SD of at least three independent experiments. B, Synergistic effects of PDPK1 inhibitors (GSK2334470 and BX795) and docetaxel in DU145, PC3 and LNCaP cells. PCa cells were treated with docetaxel and/or PDPK1 inhibitors for 72 h. The dose‐response surface curves with HSA synergy/antagonism of the combinations were generated by Combenefit software.^26^ A colour scale bar is used to indicate the level of synergism (blue) or antagonism (red) for each combination. All experiments were conducted at least three times

Since PDPK1 up‐regulation and activation have been recently shown to confer chemoresistance in breast,^45^ glioblastoma,^46^ neuroblastoma^47^ and pancreatic cancer cells,^48^ we asked whether inhibition of PDPK1 might enhance chemotherapy sensitivity in PCa cells. To test this hypothesis, we first evaluated the effects of docetaxel, a commonly used chemotherapeutic agent for PCa,^49^ and PDPK1 inhibitors (GSK2334470 and BX795) on a panel of PCa cells (DU145, PC3 and LNCaP). As shown in Figure 5A and Table S5, the AR‐positive LNCaP was more sensitive to docetaxel (IC_50_ of 7.49 ± 2.45 nM) as compared to the AR‐negative DU145 and PC3 (IC_50_ of 20.00 ± 5.68 nM and > 100 nM, respectively). In contrast, both DU145 and PC3 were more sensitive to GSK2334470 (IC_50_ of 12.74 ± 1.22 µM and 9.16 ± 0.59 µM, respectively) and BX795 (IC_50_ of 11.49 ± 5.49 µM and 5.10 ± 0.45 µM, respectively) as compared to LNCaP (IC_50_ >100 µM), consistent with the results obtained from the PDPK1 knock‐down.

Next, we investigated whether the inhibition of PDPK1 could synergize docetaxel sensitivity in PCa cells. As shown in Figure 5B, inhibition of PDPK1 enhances docetaxel sensitivity in DU145 and PC3 but not LNCaP cells, suggesting that PDPK1 inhibitors might potentiate sensitivity of refractory PCa cells to chemotherapy (Tables 1 and 2).

**TABLE 1:** Combinatory effects of docetaxel and GSK2334470 in PCa cells

Cell lines	Doc:GSK Ratio	Combination Index (CI)			Mean ± SD	Interactions
		ED_50_	ED_75_	ED_90_		
DU145	1:250	0.591	0.479	0.424	0.498 ± 0.085	Synergism
	1:500	0.534	0.513	0.532	0.526 ± 0.011	Synergism
	1:1000	0.606	0.591	0.607	0.601 ± 0.009	Synergism
	1:2000	0.785	0.759	0.757	0.767 ± 0.016	Moderate Synergism
	1:4000	1.060	0.945	0.857	0.954 ± 0.102	Nearly additive
PC3	1:250	0.232	0.302	0.441	0.325 ± 0.106	Synergism
	1:500	0.369	0.440	0.524	0.444 ± 0.078	Synergism
	1:1000	0.478	0.467	0.456	0.467 ± 0.011	Synergism
	1:2000	0.643	0.569	0.505	0.572 ± 0.069	Synergism
	1:4000	0.964	0.746	0.577	0.763 ± 0.194	Moderate Synergism
LNCaP	1:250	1.153	1.030	0.926	1.037 ± 0.114	Nearly additive
	1:500	1.011	0.940	0.883	0.945 ± 0.064	Nearly additive
	1:1000	1.003	0.928	0.877	0.936 ± 0.063	Nearly additive
	1:2000	0.877	0.755	0.677	0.769 ± 0.101	Nearly additive
	1:4000	0.827	0.802	0.841	0.824 ± 0.020	Nearly additive

**TABLE 2:** Combinatory effects of docetaxel and BX795 in PCa cells

Cell lines	Doc:BX Ratio	Combination index (CI)			Mean ± SD	Interactions
		ED_50_	ED_75_	ED_90_		
DU145	1:250	0.522	0.277	0.151	0.317 ± 0.189	Synergism
	1:500	0.385	0.195	0.100	0.227 ± 0.145	Strong Synergism
	1:1000	0.359	0.170	0.082	0.204 ± 0.142	Strong Synergism
	1:2000	0.280	0.143	0.073	0.165 ± 0.105	Strong Synergism
	1:4000	0.256	0.134	0.071	0.154 ± 0.094	Strong Synergism
PC3	1:250	0.337	0.272	0.220	0.276 ± 0.058	Strong Synergism
	1:500	0.321	0.271	0.229	0.273 ± 0.046	Strong Synergism
	1:1000	0.344	0.279	0.226	0.283 ± 0.059	Strong Synergism
	1:2000	0.323	0.268	0.223	0.271 ± 0.050	Strong Synergism
	1:4000	0.240	0.222	0.205	0.222 ± 0.017	Strong Synergism
LNCaP	1:250	1.036	1.048	1.063	1.049 ± 0.013	Nearly additive
	1:500	1.056	1.074	1.097	1.076 ± 0.021	Nearly additive
	1:1000	1.033	0.930	0.846	0.936 ± 0.094	Nearly additive
	1:2000	1.000	0.905	0.833	0.913 ± 0.084	Nearly additive
	1:4000	0.991	0.905	0.880	0.925 ± 0.058	Nearly additive

## 4 DISCUSSION

In this study, we identified 45 kinases that mediate PCa cell survival. These include previously identified proto‐oncogene such as *ERBB3*, *ERBB4*, *RET*, *SRC* and *YES1*; and pro‐survival genes involved in the PI3K and MAPK signalling such as *AKT1*, *AKT3*, *GSK3A*, *GSK3B*, *PDPK1*, *SGK3*, *MAP3K4*, *MAPK1* (also known as *ERK2*), *MAPK4* (also known as *ERK4*), *MAPK13*, *MAPK15* and *ROCK2*.^27^, ^28^, ^29^, ^30^, ^31^, ^32^, ^33^, ^34^, ^35^, ^36^, ^37^, ^38^, ^39^, ^40^ Particularly, knock‐down of PDPK1 induced tumour‐specific cell death in both DU145 and PC3 AR‐negative cells, but not in the AR‐positive LNCaP cells nor in the RWPE‐1 non‐transformed prostate epithelial cells.

Amplification or overexpression of PDPK1 has been implicated in tumourigenesis and cancer cells survival in many human cancers including breast cancer,^50^, ^51^ oesophageal squamous cell carcinoma,^52^ melanoma,^53^ gastric carcinoma,^54^ hepatocellular carcinoma ^55^ and acute myeloid leukaemia.^56^ A recent study identified the locus containing PDPK1 gene (16p13.3) is more frequently amplified in lymph node metastases and CRPC, compared to primary tumours, suggesting PDPK1 may also support cancer metastasis.^41^

PDPK1 is known to function downstream of PI3K and is required for the full activation of AKT serine/threonine kinase 1 (AKT1) and other AGC kinases such as protein kinase C (PKC), p70 ribosomal protein S6 kinase (S6K), p90 ribosomal protein S6 kinase (RSK), polo‐like kinase 1 (PLK1) and serum glucocorticoid‐dependent kinase (SGK).^57^

Upon activation, PDPK1 binds to phosphatidylinositol 3,4,5 trisphosphate (PIP3), the product of PI3K, via its pleckstrin homology (PH) domain at the plasma membrane. This in turn leads to the phosphorylation AKT at T308 and activates AKT signalling.^42^, ^58^ In the case of PDPK1 substrates that do not possess a PH domain (eg S6K, RSK and SGK), PDPK1 can still interact and bind to the hydrophobic motif of the target kinase via the PDK1‐interacting fragment (PIF) to activate the downstream signalling pathways.^40^, ^56^

In an effort to define the mechanisms of PDPK1‐mediated regulation of PCa cell survival, we observed that depletion of PDPK1 reduced SGK3 phosphorylation, but have no effects on AKT phosphorylation in cell line models with low‐ and hyperactivated background levels of AKT activity (DU145 and PC3 respectively).^59^ Furthermore, ectopic expression of a constitutively active SGK3 significantly abrogated the apoptotic effects induced by PDPK1 depletion, while no such effects were observed in cells expressing a myristoylated AKT. These data are consistent with accumulating evidence indicating that PDPK1 can contribute to cancer through activation of SGK3, independent of AKT.^58^, ^60^ For instance, it was reported that human cancers with PIK3CA mutations where AKT activity is deficient, SGK3 serves as the main PDPK1 effector to drive tumour cells survival.^43^ Similarly, SGK3 was also found to be a key mediator of PDPK1‐dependent melanomagenesis and a driver for tumour formation in breast cancer cells in both PIK3CA wild‐type and mutated cells in an AKT‐independent manner.^61^, ^62^, ^63^ Thus, our data strongly suggest that PDPK1 is mediating the survival of the AR‐negative DU145 and PC3 PCa cells through activation of SGK3, independent of AKT signalling.

Finally, our study further demonstrates that pharmacological inhibition of PDPK1, using 2 distinct chemical compounds (GSK2334470 and BX795), strongly reduced cancer cell growth in the AR‐negative DU145 and PC3 PCa cells, but not in the AR‐positive LNCaP or the RWPE‐1 non‐transformed prostate epithelial cells (data not shown). Importantly, we also report that both GSK2334470 and BX795 synergize docetaxel sensitivity in DU145 and PC3 cells, but not in LNCaP cells. Indeed, it has been observed that SGK3 can substitute for AKT in activating mTORC1,^64^ which in turn has been implicated in docetaxel resistance in PCa.^65^ To date, the role of PDPK1 in driving cancer and chemoresistance has been outlined in multiple cancers, including acute myeloid leukaemia, breast cancer and ovarian cancer.^44^ These findings further underscore the potential of PDPK1 as a therapeutic target, as they indicate that PDPK1 or SGK3 can act as druggable targets in the treatment of hormone‐refractory PCa as single agents or in combination with chemotherapeutics as components of a multitargeted therapy regimen.

## 5 CONCLUSIONS

In conclusion, we identified PDPK1 as a novel potential therapeutic target in PCa and demonstrated PDPK1 is mediating PCa cells’ survival through activation of SGK3 in an AKT‐independent manner. Our data further suggest that combination of PDPK1 inhibitors with docetaxel enhances their anti‐cancer activity, possibly by targeting SGK3‐dependent resistance mechanisms. Together, our results provide a strong rationale to investigate further the use of PDPK1 inhibitors in as novel therapeutic strategies for refractory PCa patients.

## AUTHORS’ CONTRIBUTION

IC, COL and AHAR designed the study. GN, FFLC, CWM, LWH, KKC and WML developed the methodology, collected the data and performed the analysis. GN, COL and IC wrote the manuscript. All authors reviewed and approved the manuscript. Geetha Nalairndran: Data curation (equal); Formal analysis (equal); Investigation (equal); Methodology (equal). Azad Hassan Abdul Razack: Conceptualization (equal); Funding acquisition (equal); Project administration (equal); Supervision (equal). Chun‐Wai Mai: Investigation (equal); Methodology (equal); Supervision (equal); Validation (equal). Felicia Fei‐Lei Chung: Investigation (equal); Methodology (equal); Writing‐review & editing (equal). Kok‐Keong Chan: Funding acquisition (equal); Project administration (equal); Supervision (equal). Ling‐Wei Hii: Data curation (equal); Formal analysis (equal); Investigation (equal); Methodology (equal). Wei‐Meng Lim: Data curation (equal); Formal analysis (equal); Investigation (equal); Methodology (equal). Ivy Chung: Conceptualization (equal); Funding acquisition (equal); Investigation (equal); Methodology (equal); Project administration (equal); Validation (equal); Writing‐original draft (equal); Writing‐review & editing (equal). Chee‐Onn Leong: Conceptualization (equal); Data curation (equal); Formal analysis (equal); Funding acquisition (equal); Investigation (equal); Methodology (equal); Project administration (equal); Supervision (equal); Validation (equal); Visualization (equal); Writing‐original draft (equal); Writing‐review & editing (equal).

## CONFLICT OF INTEREST

The author(s) declare no competing interests. Where authors are identified as personnel of the International Agency for Research on Cancer/World Health Organization, the authors alone are responsible for the views expressed in this article and they do not necessarily represent the decisions, policy or views of the International Agency for Research on Cancer/World Health Organization.

## DATA STATEMENT

The data that supports the findings of this study are available in the supplementary material of this article.

## Supporting information

Figures S1‐S3

Tables S1‐S5

Figure Legends

## References

[1] Bray F , Ferlay J , Soerjomataram I , Siegel RL , Torre LA , Jemal A . Global cancer statistics 2018: GLOBOCAN estimates of incidence and mortality worldwide for 36 cancers in 185 countries. CA Cancer J Clin. 2018;68:394‐424. 30207593 10.3322/caac.21492

[2] Wade CA , Kyprianou N . Profiling prostate cancer therapeutic resistance. Int J Mol Sci. 2018;19(3):904. 10.3390/ijms19030904 PMC5877765 29562686

[3] Li Q , Deng Q , Chao HP , et al. Linking prostate cancer cell AR heterogeneity to distinct castration and enzalutamide responses. Nat Commun. 2018;9:3600. 30190514 10.1038/s41467-018-06067-7 PMC6127155

[4] Chang KH , Li R , Papari‐Zareei M , et al. Dihydrotestosterone synthesis bypasses testosterone to drive castration‐resistant prostate cancer. Proc Natl Acad Sci USA. 2011;108:13728‐13733. 21795608 10.1073/pnas.1107898108 PMC3158152

[5] Sharma NL , Massie CE , Ramos‐Montoya A , et al. The androgen receptor induces a distinct transcriptional program in castration‐resistant prostate cancer in man. Cancer Cell. 2013;23:35‐47. 23260764 10.1016/j.ccr.2012.11.010

[6] Mulholland DJ , Tran LM , Li Y , et al. Cell autonomous role of PTEN in regulating castration‐resistant prostate cancer growth. Cancer Cell. 2011;19:792‐804. 21620777 10.1016/j.ccr.2011.05.006 PMC3157296

[7] Carver BS , Chapinski C , Wongvipat J , et al. Reciprocal feedback regulation of PI3K and androgen receptor signaling in PTEN‐deficient prostate cancer. Cancer Cell. 2011;19:575‐586. 21575859 10.1016/j.ccr.2011.04.008 PMC3142785

[8] Kumar A , Coleman I , Morrissey C , et al. Substantial interindividual and limited intraindividual genomic diversity among tumors from men with metastatic prostate cancer. Nat Med. 2016;22:369‐378. 26928463 10.1038/nm.4053 PMC5045679

[9] Niu Y , Altuwaijri S , Lai KP , et al. Androgen receptor is a tumor suppressor and proliferator in prostate cancer. Proc Natl Acad Sci USA. 2008;105:12182‐12187. 18723679 10.1073/pnas.0804700105 PMC2527886

[10] He Y , Hooker E , Yu EJ , et al. Androgen signaling is essential for development of prostate cancer initiated from prostatic basal cells. Oncogene. 2019;38:2337‐2350. 30510232 10.1038/s41388-018-0583-7 PMC6440846

[11] Sampson N , Neuwirt H , Puhr M , Klocker H , Eder IE . In vitro model systems to study androgen receptor signaling in prostate cancer. Endocr Relat Cancer. 2013;20:R49‐64. 23447570 10.1530/ERC-12-0401

[12] Konig R , Chiang CY , Tu BP , et al. A probability‐based approach for the analysis of large‐scale RNAi screens. Nat Methods. 2007;4:847‐849. 17828270 10.1038/nmeth1089

[13] Tiong KH , Tan BS , Choo HL , et al. Fibroblast growth factor receptor 4 (FGFR4) and fibroblast growth factor 19 (FGF19) autocrine enhance breast cancer cells survival. Oncotarget. 2016;7:57633‐57650. 27192118 10.18632/oncotarget.9328 PMC5295378

[14] Hii LW , Chung FF , Soo JS , Tan BS , Mai CW , Leong CO . Histone deacetylase (HDAC) inhibitors and doxorubicin combinations target both breast cancer stem cells and non‐stem breast cancer cells simultaneously. Breast Cancer Res Treat. 2020;179:615‐629. 31784862 10.1007/s10549-019-05504-5

[15] Hii LW , Chung FF , Mai CW , et al. Sphingosine kinase 1 regulates the survival of breast cancer stem cells and non‐stem breast cancer cells by suppression of STAT1. Cells. 2020;9:886. 10.3390/cells9040886 PMC7226795 32260399

[16] Ibrahim N , He L , Leong CO , et al. BRCA1‐associated epigenetic regulation of p73 mediates an effector pathway for chemosensitivity in ovarian carcinoma. Can Res. 2010;70:7155‐7165. 10.1158/0008-5472.CAN-10-0668 PMC2940979 20807817

[17] Rocco JW , Leong CO , Kuperwasser N , DeYoung MP , Ellisen LW . p63 mediates survival in squamous cell carcinoma by suppression of p73‐dependent apoptosis. Cancer Cell. 2006;9:45‐56. 16413471 10.1016/j.ccr.2005.12.013

[18] Tan BS , Tiong KH , Muruhadas A , et al. CYP2S1 and CYP2W1 mediate 2‐(3,4‐dimethoxyphenyl)‐5‐fluorobenzothiazole (GW‐610, NSC 721648) sensitivity in breast and colorectal cancer cells. Mol Cancer Ther. 2011;10:1982‐1992. 21831963 10.1158/1535-7163.MCT-11-0391

[19] Rist S , Carney Almroth B , Hartmann NB , Karlsson TM . A critical perspective on early communications concerning human health aspects of microplastics. Elsevier B.V. 2018;626:720‐726. 10.1016/j.scitotenv.2018.01.092 29396337

[20] Soo HC , Chung FFL , Lim KH , et al. Cudraflavone C induces tumor‐specific apoptosis in colorectal cancer cells through inhibition of the phosphoinositide 3‐kinase (PI3K)‐AKT pathway. PLoS One. 2017;12:e0170551. 28107519 10.1371/journal.pone.0170551 PMC5249192

[21] Chou T‐C . Drug combination studies and their synergy quantification using the Chou‐Talalay method. Can Res. 2010;70:440‐446. 10.1158/0008-5472.CAN-09-1947 20068163

[22] Chou TC , Talalay P . Quantitative analysis of dose‐effect relationships: the combined effects of multiple drugs or enzyme inhibitors. Adv Enzyme Regul. 1984;22:27‐55. 6382953 10.1016/0065-2571(84)90007-4

[23] Stone EL , Citossi F , Singh R , et al. Antitumour benzothiazoles. Part 32: DNA adducts and double strand breaks correlate with activity; synthesis of 5F203 hydrogels for local delivery. Bioorg Med Chem. 2015;23:6891‐6899. 26474663 10.1016/j.bmc.2015.09.052

[24] Al‐Khdhairawi AAQ , Krishnan P , Mai CW , et al. A Bis‐benzopyrroloisoquinoline Alkaloid Incorporating a Cyclobutane Core and a Chlorophenanthroindolizidine Alkaloid with Cytotoxic Activity from Ficus fistulosa var. tengerensis. J Nat Prod. 2017;80:2734‐2740. 28926237 10.1021/acs.jnatprod.7b00500

[25] Voon YL , Ahmad M , Wong PF , et al. Nutlin‐3 sensitizes nasopharyngeal carcinoma cells to cisplatin‐induced cytotoxicity. Oncol Rep. 2015;34:1692‐1700. 26252575 10.3892/or.2015.4177 PMC4564086

[26] Di Veroli GY , Fornari C , Wang D , et al. Combenefit: an interactive platform for the analysis and visualization of drug combinations. Bioinformatics. 2016;32:2866‐2868. 27153664 10.1093/bioinformatics/btw230 PMC5018366

[27] Sasaki T , Nakashiro K , Tanaka H , et al. Knockdown of Akt isoforms by RNA silencing suppresses the growth of human prostate cancer cells in vitro and in vivo. Biochem Biophys Res Comm. 2010;399:79‐83. 20638364 10.1016/j.bbrc.2010.07.045

[28] Cariaga‐Martinez AE , Lopez‐Ruiz P , Nombela‐Blanco MP , et al. Distinct and specific roles of AKT1 and AKT2 in androgen‐sensitive and androgen‐independent prostate cancer cells. Cell Signal. 2013;25:1586‐1597. 23567263 10.1016/j.cellsig.2013.03.019

[29] Wang W , Shen T , Dong B , et al. MAPK4 overexpression promotes tumor progression via noncanonical activation of AKT/mTOR signaling. J Clin Investig. 2019;129:1015‐1029. 30688659 10.1172/JCI97712 PMC6391107

[30] Gao F , Al‐Azayzih A , Somanath PR . Discrete functions of GSK3alpha and GSK3beta isoforms in prostate tumor growth and micrometastasis. Oncotarget. 2015;6:5947‐5962. 25714023 10.18632/oncotarget.3335 PMC4467413

[31] Darrington RS , Campa VM , Walker MM , et al. Distinct expression and activity of GSK‐3alpha and GSK‐3beta in prostate cancer. Int J Cancer. 2012;131:E872‐E883. 22539113 10.1002/ijc.27620

[32] Mulholland DJ , Dedhar S , Wu H , Nelson CC . PTEN and GSK3beta: key regulators of progression to androgen‐independent prostate cancer. Oncogene. 2006;25:329‐337. 16421604 10.1038/sj.onc.1209020

[33] Liao X , Thrasher JB , Holzbeierlein J , Stanley S , Li B . Glycogen synthase kinase‐3beta activity is required for androgen‐stimulated gene expression in prostate cancer. Endocrinology. 2004;145:2941‐2949. 14988390 10.1210/en.2003-1519

[34] Schutz SV , Schrader AJ , Zengerling F , Genze F , Cronauer MV , Schrader M . Inhibition of glycogen synthase kinase‐3beta counteracts ligand‐independent activity of the androgen receptor in castration resistant prostate cancer. PLoS One. 2011;6:e25341. 21980429 10.1371/journal.pone.0025341 PMC3183056

[35] Zhu Q , Yang J , Han S , et al. Suppression of glycogen synthase kinase 3 activity reduces tumor growth of prostate cancer in vivo. Prostate. 2011;71:835‐845. 21456066 10.1002/pros.21300

[36] Imada K , Shiota M , Kohashi K , et al. Mutual regulation between Raf/MEK/ERK signaling and Y‐box‐binding protein‐1 promotes prostate cancer progression. Clin Cancer Res. 2013;19:4638‐4650. 23838318 10.1158/1078-0432.CCR-12-3705

[37] Hong SK , Kim JH , Lin MF , Park JI . The Raf/MEK/extracellular signal‐regulated kinase 1/2 pathway can mediate growth inhibitory and differentiation signaling via androgen receptor downregulation in prostate cancer cells. Exp Cell Res. 2011;317:2671‐2682. 21871886 10.1016/j.yexcr.2011.08.008 PMC3189339

[38] Gong H , Zhou L , Khelfat L , et al. Rho‐associated protein kinase (ROCK) promotes proliferation and migration of PC‐3 and DU145 prostate cancer cells by targeting LIM kinase 1 (LIMK1) and matrix metalloproteinase‐2 (MMP‐2). Medical Sci Mon. 2019;25:3090‐3099. 10.12659/MSM.912098 PMC6500105 31026254

[39] Liu H , Hou T , Ju W , Xing Y , Zhang X , Yang J . MicroRNA122 downregulates Rhoassociated protein kinase 2 expression and inhibits the proliferation of prostate carcinoma cells. Mol Med Rep. 2019;19:3882‐3888. 30816534 10.3892/mmr.2019.9995

[40] Kroiss A , Vincent S , Decaussin‐Petrucci M , et al. Androgen‐regulated microRNA‐135a decreases prostate cancer cell migration and invasion through downregulating ROCK1 and ROCK2. Oncogene. 2015;34:2846‐2855. 25065599 10.1038/onc.2014.222

[41] Choucair KA , Guerard KP , Ejdelman J , et al. The 16p13.3 (PDPK1) genomic gain in prostate cancer: A potential role in disease progression. Transl Oncol. 2012;5:453‐460. 23401739 10.1593/tlo.12286 PMC3568696

[42] Gagliardi PA , Puliafito A , Primo L . PDK1: At the crossroad of cancer signaling pathways. Semin Cancer Biol. 2018;48:27‐35. 28473254 10.1016/j.semcancer.2017.04.014

[43] Vasudevan KM , Barbie DA , Davies MA , et al. AKT‐independent signaling downstream of oncogenic PIK3CA mutations in human cancer. Cancer Cell. 2009;16:21‐32. 19573809 10.1016/j.ccr.2009.04.012 PMC2752826

[44] Emmanouilidi A , Falasca M . Targeting PDK1 for chemosensitization of cancer cells. Cancers. 2017;9. 10.3390/cancers9100140 PMC5664079 29064423

[45] Castel P , Ellis H , Bago R , et al. PDK1‐SGK1 signaling sustains AKT‐independent mTORC1 activation and confers resistance to PI3Kalpha inhibition. Cancer Cell. 2016;30:229‐242. 27451907 10.1016/j.ccell.2016.06.004 PMC4982440

[46] Velpula KK , Guda MR , Sahu K , et al. Metabolic targeting of EGFRvIII/PDK1 axis in temozolomide resistant glioblastoma. Oncotarget. 2017;8:35639‐35655. 28410193 10.18632/oncotarget.16767 PMC5482605

[47] Qi L , Toyoda H , Xu DQ , et al. PDK1‐mTOR signaling pathway inhibitors reduce cell proliferation in MK2206 resistant neuroblastoma cells. Cancer Cell Int. 2015;15:91. 26421002 10.1186/s12935-015-0239-4 PMC4587771

[48] Li D , Mullinax JE , Aiken T , et al. Loss of PDPK1 abrogates resistance to gemcitabine in label‐retaining pancreatic cancer cells. BMC Cancer. 2018;18:772. 30064387 10.1186/s12885-018-4690-1 PMC6069886

[49] Vale CL , Burdett S , Rydzewska LHM , et al. Addition of docetaxel or bisphosphonates to standard of care in men with localised or metastatic, hormone‐sensitive prostate cancer: a systematic review and meta‐analyses of aggregate data. Lancet Oncol. 2016;17:243‐256. 26718929 10.1016/S1470-2045(15)00489-1 PMC4737894

[50] Mihaly Z , Kormos M , Lanczky A , et al. A meta‐analysis of gene expression‐based biomarkers predicting outcome after tamoxifen treatment in breast cancer. Breast Cancer Res Treat. 2013;140:219‐232. 23836010 10.1007/s10549-013-2622-y

[51] Maurer M , Su T , Saal LH , et al. 3‐Phosphoinositide‐dependent kinase 1 potentiates upstream lesions on the phosphatidylinositol 3‐kinase pathway in breast carcinoma. Can Res. 2009;69:6299‐6306. 10.1158/0008-5472.CAN-09-0820 PMC2727605 19602588

[52] Yang Z , Wu Z , Liu T , et al. Upregulation of PDK1 associates with poor prognosis in esophageal squamous cell carcinoma with facilitating tumorigenicity in vitro. Med Oncol. 2014;31:337. 25416048 10.1007/s12032-014-0337-5

[53] Scortegagna M , Ruller C , Feng Y , et al. Genetic inactivation or pharmacological inhibition of Pdk1 delays development and inhibits metastasis of Braf(V600E):Pten(‐/‐) melanoma. Oncogene. 2014;33:4330‐4339. 24037523 10.1038/onc.2013.383 PMC3955742

[54] Bai X , Li P , Xie Y , et al. Overexpression of 3‐phosphoinositide‐dependent protein kinase‐1 is associated with prognosis of gastric carcinoma. Tumour Biol. 2016;37:2333‐2339. 26373731 10.1007/s13277-015-4024-8

[55] Wang J , Liu F , Ao P , et al. Correlation of PDK1 expression with clinicopathologic features and prognosis of hepatocellular carcinoma. OncoTargets and Therapy. 2016;9:5597‐5602. 27672330 10.2147/OTT.S110646 PMC5024765

[56] Zabkiewicz J , Pearn L , Hills RK , et al. The PDK1 master kinase is over‐expressed in acute myeloid leukemia and promotes PKC‐mediated survival of leukemic blasts. Haematologica. 2014;99:858‐864. 24334295 10.3324/haematol.2013.096487 PMC4008098

[57] Pearce LR , Komander D , Alessi DR . The nuts and bolts of AGC protein kinases. Nat Rev Mol Cell Biol. 2010;11:9‐22. 20027184 10.1038/nrm2822

[58] Di Blasio L , Gagliardi PA , Puliafito A , Primo L . Serine/threonine kinase 3‐phosphoinositide‐dependent protein kinase‐1 (PDK1) as a key regulator of cell migration and cancer dissemination. Cancers. 2017;9. 10.3390/cancers9030025 PMC5366820 28287465

[59] Nogueira V , Patra KC , Hay N . Selective eradication of cancer displaying hyperactive Akt by exploiting the metabolic consequences of Akt activation. eLife. 2018;7:e32213. 29687779 10.7554/eLife.32213 PMC5980228

[60] Lien EC , Dibble CC , Toker A . PI3K signaling in cancer: beyond AKT. Curr Opin Cell Biol. 2017;45:62‐71. 28343126 10.1016/j.ceb.2017.02.007 PMC5482768

[61] Scortegagna M , Lau E , Zhang T , et al. PDK1 and SGK3 contribute to the growth of BRAF‐mutant melanomas and are potential therapeutic targets. Can Res. 2015;75:1399‐1412. 10.1158/0008-5472.CAN-14-2785 PMC4383687 25712345

[62] Silva JM , Bulman C , McMahon M . BRAFV600E cooperates with PI3K signaling, independent of AKT, to regulate melanoma cell proliferation. Molecular cancer research: MCR. 2014;12:447‐463. 24425783 10.1158/1541-7786.MCR-13-0224-T PMC3966216

[63] Gagliardi PA , di Blasio L , Orso F , et al. 3‐phosphoinositide‐dependent kinase 1 controls breast tumor growth in a kinase‐dependent but Akt‐independent manner. Neoplasia. 2012;14:719‐731. 22952425 10.1593/neo.12856 PMC3431179

[64] Bago R , Sommer E , Castel P , et al. The hVps34‐SGK3 pathway alleviates sustained PI3K/Akt inhibition by stimulating mTORC1 and tumour growth. EMBO J. 2016;35:1902‐1922. 27481935 10.15252/embj.201693929 PMC5007552

[65] Qian DZ , Rademacher BL , Pittsenbarger J , et al. CCL2 is induced by chemotherapy and protects prostate cancer cells from docetaxel‐induced cytotoxicity. Prostate. 2010;70:433‐442. 19866475 10.1002/pros.21077 PMC2931415

