# Erratum

**DOI:** 10.1111/jcmm.16112

**Published:** 2020-12-08

In Nalairndran et al,^1^ the published article contains errors in Figures 1, 2, 3. The correct figures are shown below. The authors confirm all results and conclusions of this article remain unchanged.

**Figure 1:**
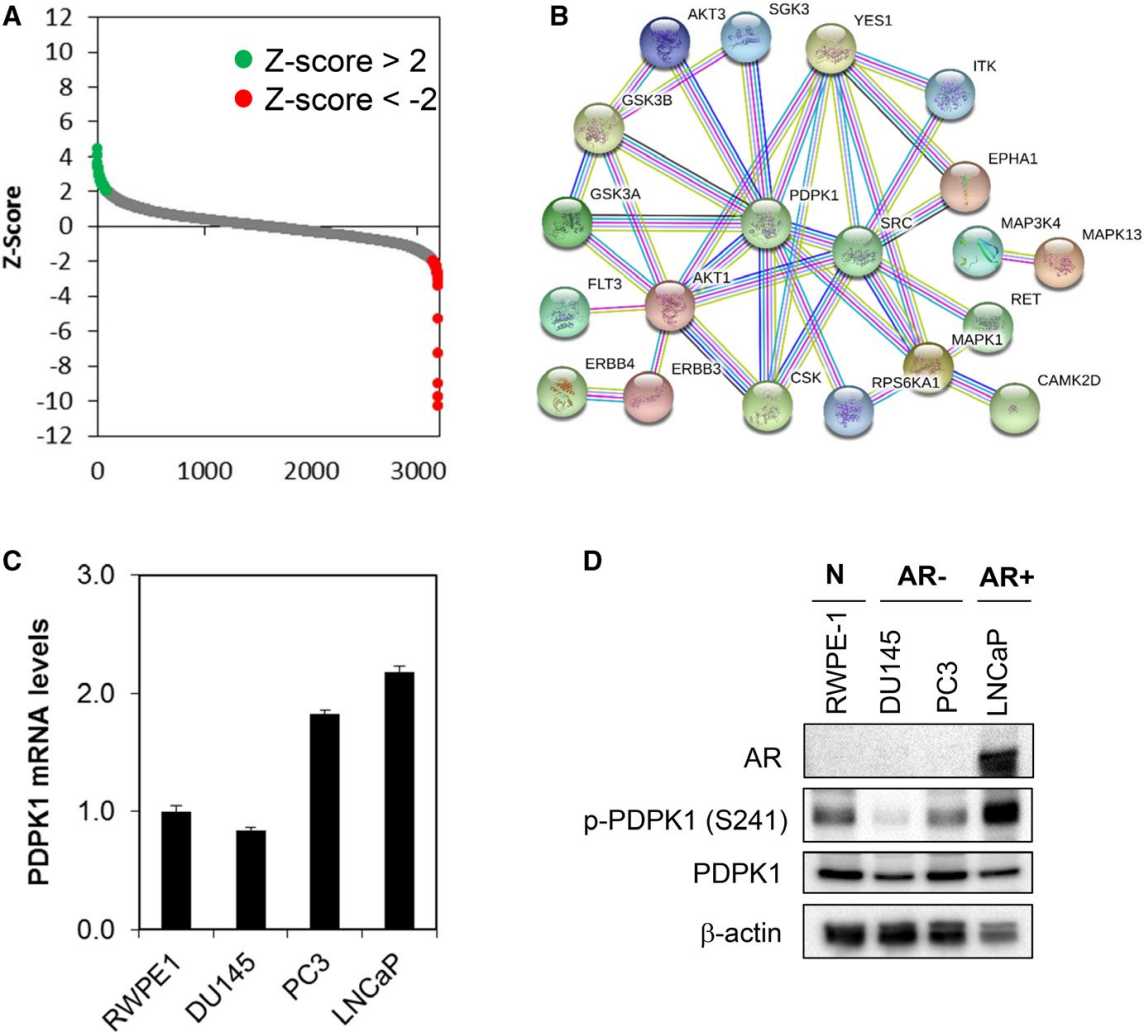
Kinome‐wide shRNA library screen identifies PDPK1 as putative target regulating the survival of PCa cells. A, Kinase shRNA screen scatter plot. *Z*‐scores are plotted on the *y*‐axis against 3109 corresponding shRNAs on the *x*‐axis. The red and green circled dots represent shRNA hits, which the former inhibited cell proliferation and the latter promoted cell proliferation. B, Protein‐protein interaction network of the PDPK1 target proteins. C and D, PDPK1 is expressed in a subset of PCa cells and RWPE 1 non‐transformed prostate epithelial cells. PDPK1 mRNA expression was evaluated by qPCR with GAPDH as housekeeping gene. The level of PDPK1 protein expression was detected by immunoblotting with β‐actin as loading control

**Figure 2:**
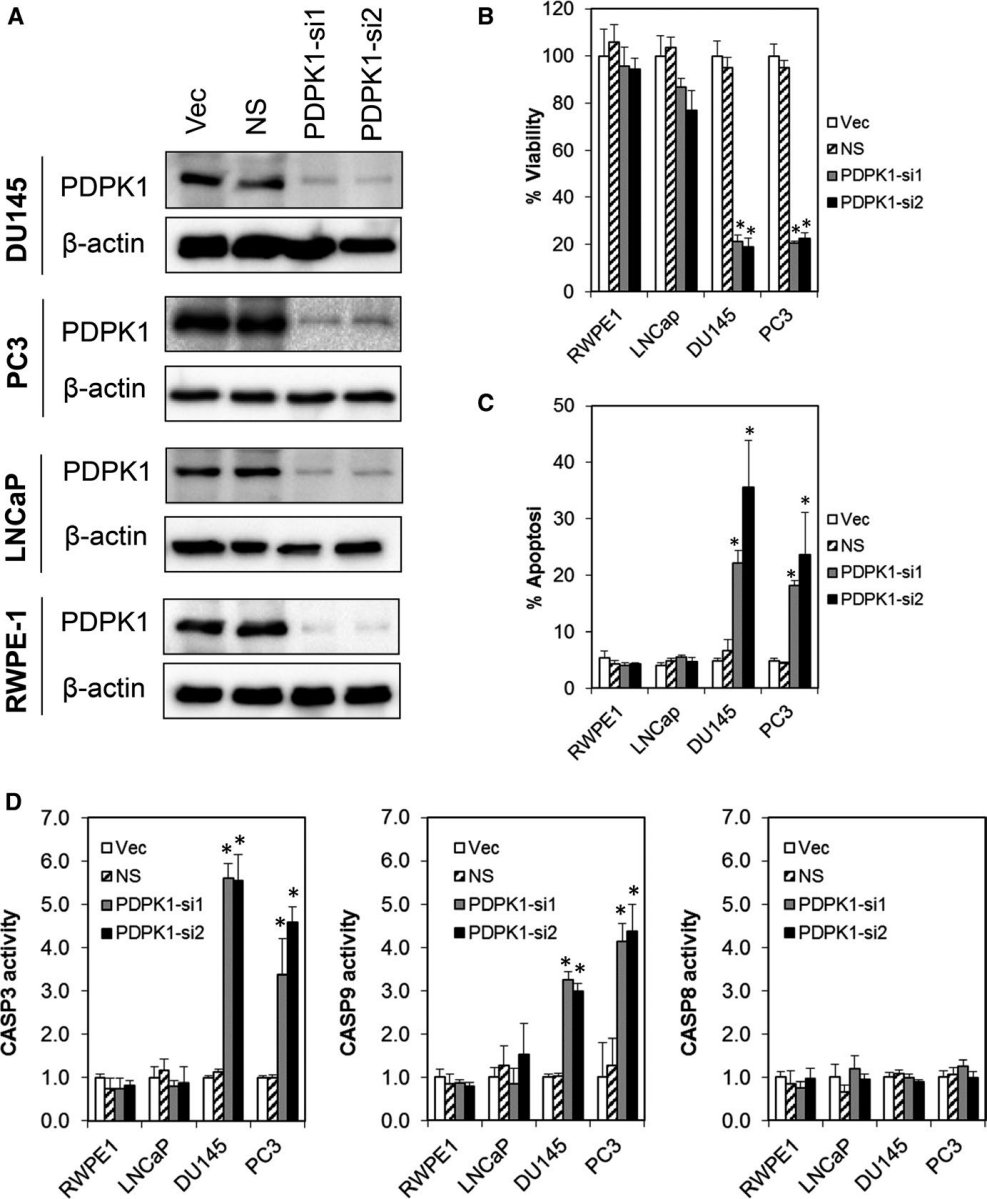
Depletion of endogenous PDPK1 induces tumour‐specific cell death in PCa cells. A, Effective PDPK1 knock‐down was achieved by two independent shRNA constructs targeting PDPK1 (PDPK1‐si1 and PDPK1‐si2). Lysates were harvested at 72 h post‐lentiviral transduction and analysed by immunoblotting. B and C, PDPK1 depletion selectively inhibited the proliferation and induced apoptosis in AR‐negative DU145 and PC3 PCa cells but not in AR‐positive LNCaP or RWPE‐1 non‐transformed prostate epithelial cells. Cell viability and apoptosis were measured using CellTiter‐Glo^®^ assay and annexin V/7‐AAD flow cytometry at 72 h post‐transduction. D, Depletion of endogenous PDPK1 induced caspase 3 and 9 activities. Caspase 3, 8 and 9 activities were evaluated by CaspaseGlo assay at 72 h post‐transduction. Bars represent means ± SD of three independent experiments. (*) indicates statistical significance compared with NS control cells (*P* < 0.01, Student's *t* test)

**Figure 3:**
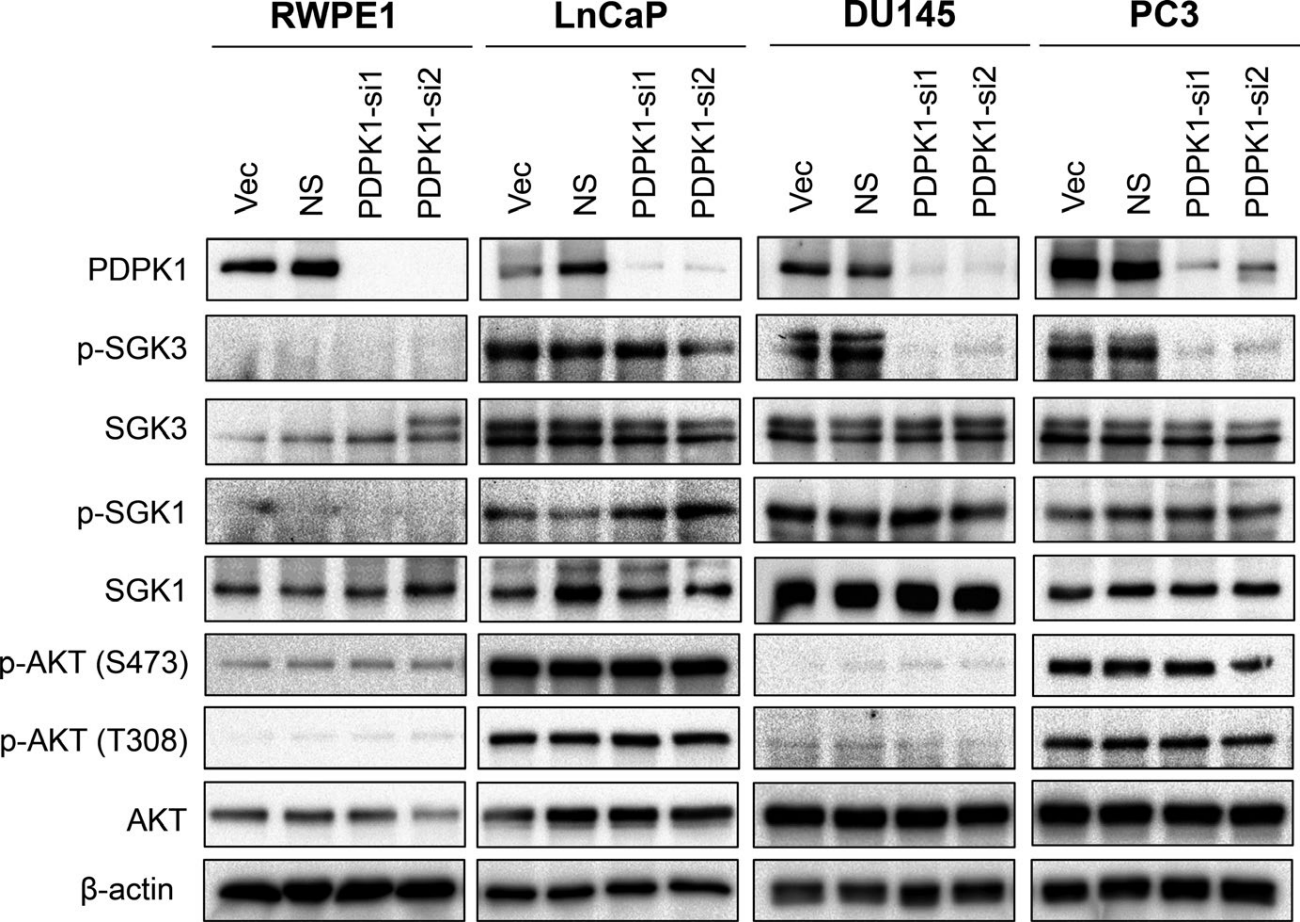
Depletion of endogenous PDPK1 reduces SGK3 phosphorylation. PDPK1 depletion down‐regulated SGK3 phosphorylation in AR‐negative DU145 and PC3 cells, but not in AR‐positive LNCaP PCa cells or RWPE‐1 non‐transformed prostate epithelial cells. The protein expression and phosphorylation of AKT, SGK1 and SGK3 Lysates were analysed by immunoblotting with β‐actin and served as loading controls
