# Supplementary material for: Phosphoinositide‐dependent Kinase‐1 (PDPK1) regulates serum/glucocorticoid‐regulated Kinase 3 (SGK3) for prostate cancer cell survival
Source: J Cell Mol Med. 2020 Sep 14;24(20):12188–98. doi: 10.1111/jcmm.15876 (PMC7578863; doi:10.1111/jcmm.15876)
Supplement: Supplementary file 1 — Figures S1‐S3 [file JCMM-24-12188-s001.pptx]

## Slide 1
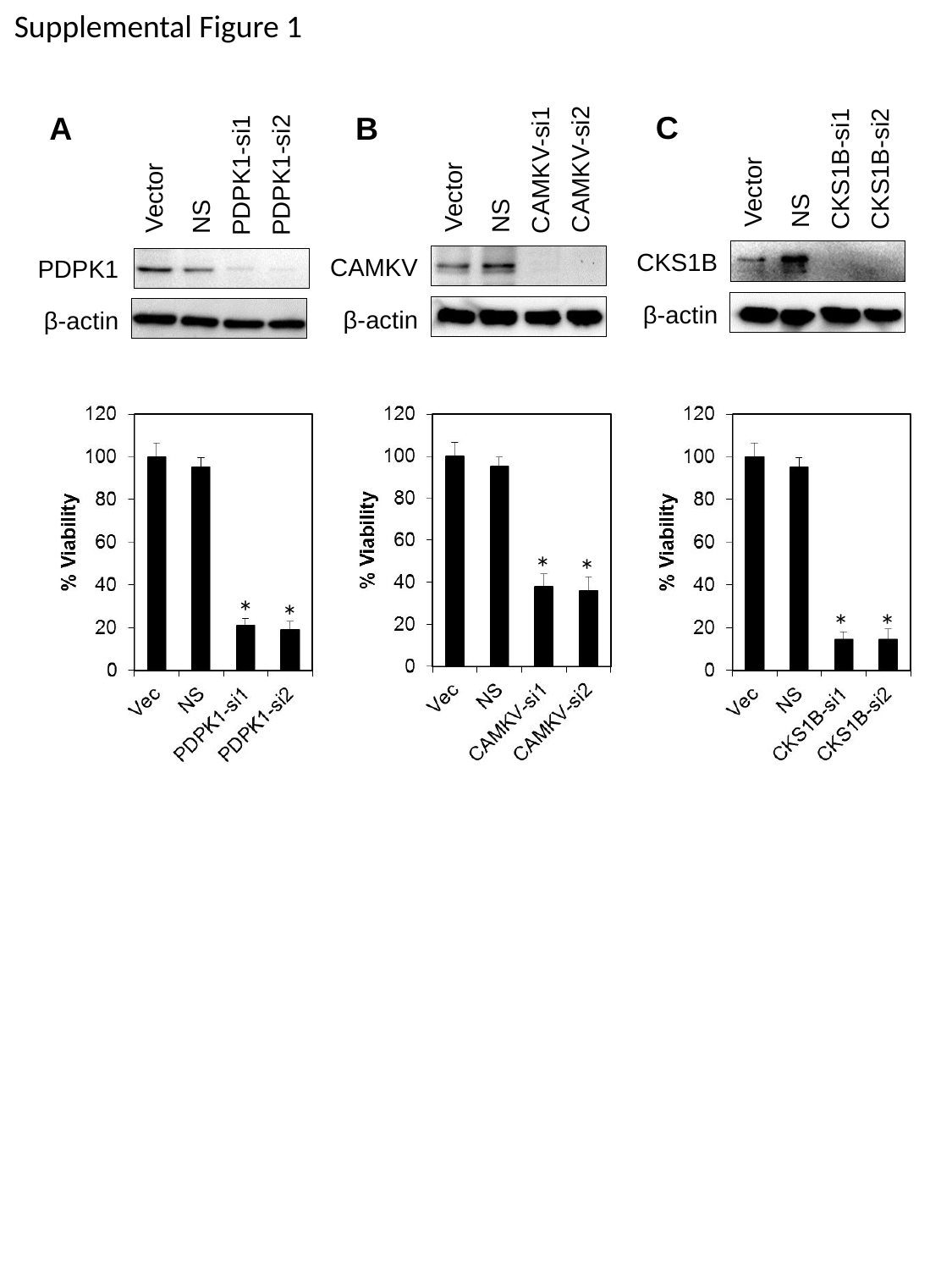

Supplemental Figure 1
C
A
B
CKS1B-si1
CAMKV-si1
CKS1B-si2
CAMKV-si2
PDPK1-si1
PDPK1-si2
Vector
Vector
Vector
NS
NS
NS
CKS1B
CAMKV
PDPK1
β-actin
β-actin
β-actin
*
*
*
*
*
*

## Slide 2
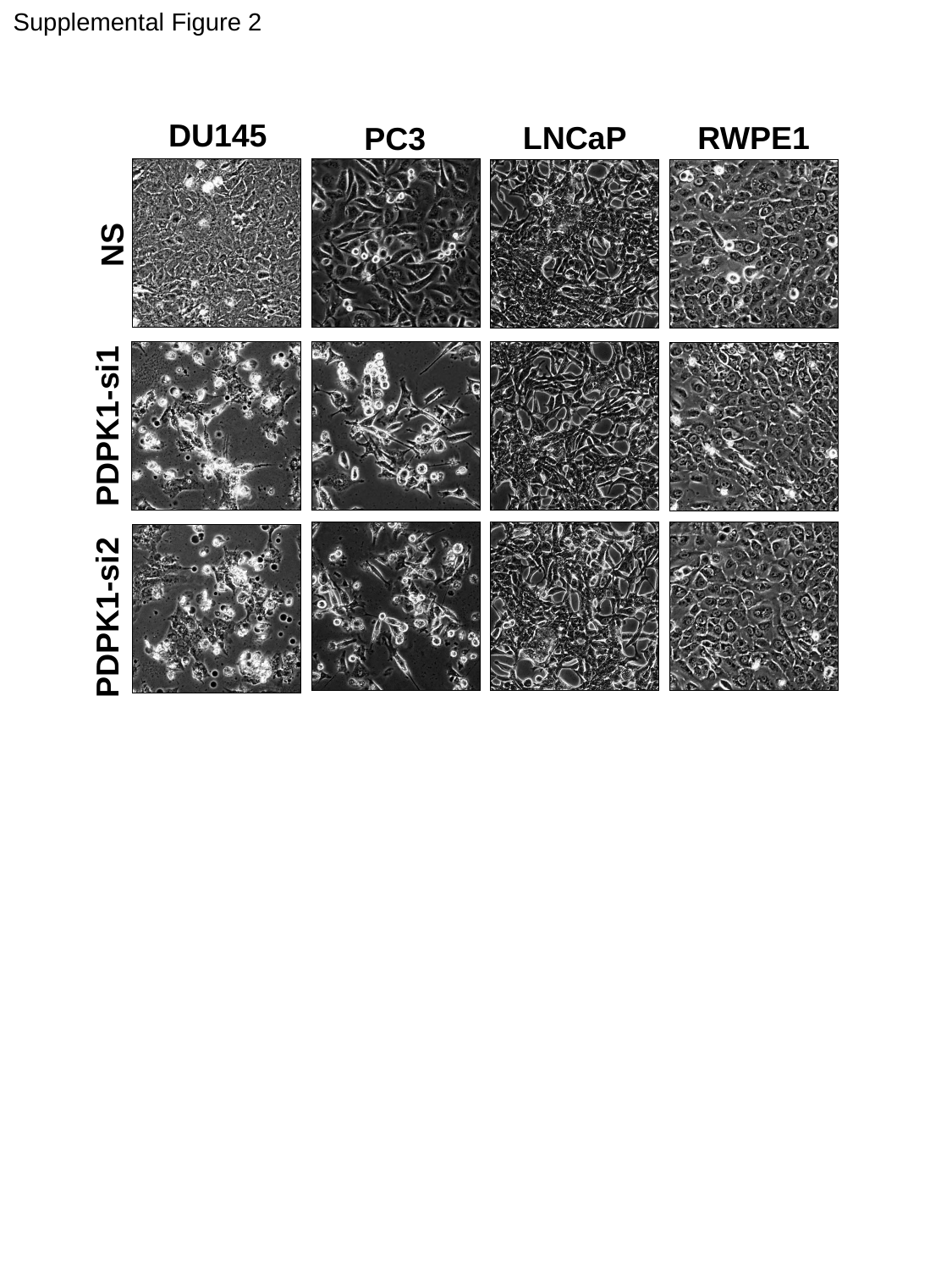

Supplemental Figure 2
DU145
LNCaP
RWPE1
PC3
NS
PDPK1-si1
PDPK1-si2

## Slide 3
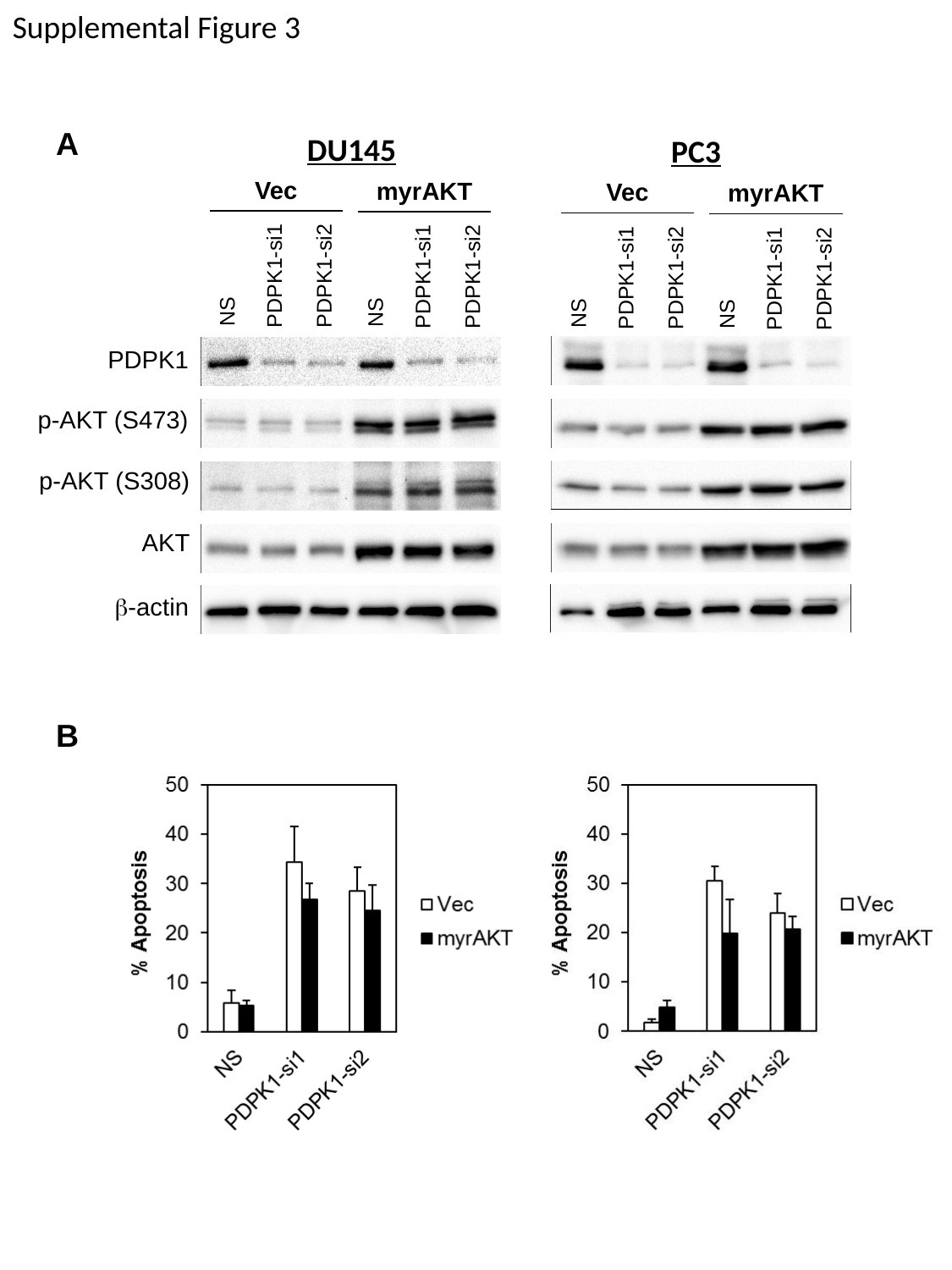

Supplemental Figure 3
A
DU145
PC3
Vec
myrAKT
Vec
myrAKT
PDPK1-si2
PDPK1-si1
PDPK1-si2
PDPK1-si1
PDPK1-si2
PDPK1-si1
PDPK1-si2
PDPK1-si1
NS
NS
NS
NS
PDPK1
p-AKT (S473)
p-AKT (S308)
AKT
β-actin
B
