# Supplementary material for: Phosphoinositide‐dependent Kinase‐1 (PDPK1) regulates serum/glucocorticoid‐regulated Kinase 3 (SGK3) for prostate cancer cell survival
Source: J Cell Mol Med. 2020 Sep 14;24(20):12188–98. doi: 10.1111/jcmm.15876 (PMC7578863; doi:10.1111/jcmm.15876)
Supplement: Supplementary file 2 — Tables S1‐S5 [file JCMM-24-12188-s002.docx]

**SUPPLMENTAL TABLE**

**Supplemental Table 1.** Primer sequences for qPCR

| **Gene** | **Forward primer** | **Reverse primer** |
| --- | --- | --- |
| PDPK1 | 5’-GGCCAGGACCACCAGCCA-3’ | 5’- AGCCAGGACAACCGTGGAAA-3’ |
| GAPDH | 5’-GTCTCCTCTGACTTCAACAGCG-3’ | 5’-ACCACCCTGTTGCTGTAGCCAA-3’ |

**Supplemental Table 2.** shRNAs target sequences

| **shRNAs** | **Target Sequence** |
| --- | --- |
| PDPK1-si1 | 5’-CAACATAGAGCAGTACATTCA-3’ |
| PDPK1-si2 | 5’-CAAAGTTCTGAAAGGTGAAAT-3’ |
| CAMKV-si1 | 5’-GATGAGGAAGTAGGGTTAAAC-3’ |
| CAMKV-si2 | 5’-GCGGAAAGCTGCCAAGAACGA-3’ |
| CKS1B-si1 | 5’-CGAGGAGTTTGAGTATCGACA-3’ |
| CKS1B-si2 | 5’-CATCTGATGTCTGAATCTGAA-3’ |

**Supplemental Table 3**. Range of Combination Index (CI)

| **CI Range** | **Description** |
| --- | --- |
| < 0.1 | Very strong synergism |
| 0.1 - 0.3 | Strong Synergism |
| 0.3 - 0.7 | Synergism |
| 0.7 - 0.85 | Moderate Synergism |
| 0.85 - 0.90 | Slight Synergism |
| 0.90 - 1.10 | Nearly additive |
| 1.10 - 1.20 | Slight antagonism |
| 1.20 - 1.45 | Moderate antagonism |
| 1.45 - 3.3 | Antagonism |
| 3.3 - 10 | Strong Antagonism |
| >10 | Very strong antagonism |

**Supplemental Table 4:** Candidate kinases that mediate survival of DU145 cells.

| **Gene Symbol** | **Gene Name** | **Z-Score** |
| --- | --- | --- |
| EPHA1 | EPH receptor A1 | -10.26 |
| CAMKV | CaM kinase-like vesicle-associated | -9.77 |
| PHKA1 | Phosphorylase kinase, alpha 1 (muscle) | -9.02 |
| PDPK1 | 3-phosphoinositide dependent protein kinase-1 | -7.30 |
| MAPK4 | Mitogen-activated protein kinase 4 | -5.31 |
| ROCK2 | Rho-associated, coiled-coil containing protein kinase 2 | -3.44 |
| STK17B | Serine/threonine kinase 17b | -3.36 |
| CKS1B | CDC28 protein kinase regulatory subunit 1B | -3.00 |
| GSK3B | Glycogen synthase kinase 3 beta | -2.82 |
| EPHA10 | EPH receptor A10 | -2.76 |
| MAP3K4 | Mitogen-activated protein kinase kinase kinase 4 | -2.66 |
| ADCK2 | aarF domain containing kinase 2 | -2.65 |
| MAST4 | Microtubule associated serine/threonine kinase family member 4 | -2.62 |
| MAPK1 | Mitogen-activated protein kinase 1 | -2.59 |
| SGK3 | Serum/glucocorticoid regulated kinase family, member 3 | -2.52 |
| NRK | Nik related kinase | -2.48 |
| MYO3A | Myosin IIIA | -2.46 |
| RPS6KA1 | Ribosomal protein S6 kinase, 90kDa, polypeptide 1 | -2.45 |
| GSK3A | Glycogen synthase kinase 3 alpha | -2.44 |
| MAPK13 | Mitogen-activated protein kinase 13 | -2.43 |
| BRD3 | Bromodomain containing 3 | -2.39 |
| MLKL | Mixed lineage kinase domain-like | -2.39 |
| FLT3 | fms-related tyrosine kinase 3 | -2.38 |
| GRK4 | G protein-coupled receptor kinase 4 | -2.31 |
| AKT3 | v-akt murine thymoma viral oncogene homolog 3 (protein kinase B, gamma) | -2.29 |
| CAMK2D | Calcium/calmodulin-dependent protein kinase II delta | -2.27 |
| NRK | Nik related kinase | -2.26 |
| NUAK1 | NUAK family, SNF1-like kinase, 1 | -2.25 |
| AKT1 | v-akt murine thymoma viral oncogene homolog 1 | -2.25 |
| ITK | IL2-inducible T-cell kinase | -2.25 |
| GSK3B | Glycogen synthase kinase 3 beta | -2.21 |
| STK16 | Serine/threonine kinase 16 | -2.21 |
| STK17B | Serine/threonine kinase 17b | -2.19 |
| CDK9 | Cyclin-dependent kinase 9 | -2.19 |
| NTRK3 | Neurotrophic tyrosine kinase, receptor, type 3 | -2.17 |
| MAPK13 | Mitogen-activated protein kinase 13 | -2.16 |
| RET | ret proto-oncogene | -2.12 |
| CDK8 | Cyclin-dependent kinase 8 | -2.11 |
| CDC42BPG | CDC42 binding protein kinase gamma (DMPK-like) | -2.11 |
| YES1 | v-yes-1 Yamaguchi sarcoma viral oncogene homolog 1 | -2.10 |
| ERBB4 | v-erb-a erythroblastic leukemia viral oncogene homolog 4 (avian) | -2.09 |
| MAPK15 | Mitogen-activated protein kinase 15 | -2.09 |
| CSK | c-src tyrosine kinase | -2.08 |
| CDC42BPA | CDC42 binding protein kinase alpha (DMPK-like) | -2.07 |
| ANKK1 | Ankyrin repeat and kinase domain containing 1 | -2.06 |

**Supplemental Table 5:** Half maximal inhibitory concentration (IC_50_) values of docetaxel and PDPK1 inhibitors against PCa cells.

| **Cell lines** | **Docetaxel (nM)** | **GSK2334470 (µM)** | **BX795 (µM)** |
| --- | --- | --- | --- |
| LNCaP | 7.49 ± 2.45 | > 100 | > 100 |
| DU145 | 20.00 ± 5.68 | 12.74 ± 1.22 | - 1. ± 5.49 |
| PC3 | > 100 | 9.16 ± 0.59 | 5.10 ± 0.45 |
