# Supplementary material for: Phosphoinositide‐dependent Kinase‐1 (PDPK1) regulates serum/glucocorticoid‐regulated Kinase 3 (SGK3) for prostate cancer cell survival
Source: J Cell Mol Med. 2020 Sep 14;24(20):12188–98. doi: 10.1111/jcmm.15876 (PMC7578863; doi:10.1111/jcmm.15876)
Supplement: Supplementary file 3 — Figure Legends [file JCMM-24-12188-s003.docx]

**SUPPLEMENTAL FIGURE LEGENDS**

**Supplemental Figure 1.** **Validation of hits identified from the primary screen.** The levels of gene knockdown were evaluated using 2 independent lentiviral shRNAs targeting PDPK1, CAMKV and CKS1B. Protein expression was determined by Western blot 72 h after transduction. Cell viability was measured by CellTiter-Glo® assay 72 h after transduction. Bars represent means ± s.d of at least three independent experiments. (*) indicates statistical significance compared with control cells transduced with a non-targeting shRNA (NS) (P < 0.01, Student’s *t*-test).

**Supplemental Figure 2.** **PDPK1 knock-down induced apoptosis in PCa cells.** Cells were treated as in Figure 2. Cell morphology was recorded at 100X magnification 72 h post-transduction.

**Supplemental Figure 3: Ectopic expression of myristoylated AKT did not rescue cell death induced by PDPK1 depletion. (A)** Cells were transiently transfected with myristoylated AKT concurrently with either NS or PDPK1-targeting shRNAs. Lysates were collected 72 h post-transfection and analysed by Western blotting. **(B)** Apoptosis was analysed by annexin V/7-AAD flowcytometry. Bars represent means ± s.d. of three independent experiments.
